# Inflammation time-axis in aseptic loosening of total knee arthroplasty: A preliminary study

**DOI:** 10.1371/journal.pone.0221056

**Published:** 2019-08-30

**Authors:** Tereza Dyskova, Eva Kriegova, Zuzana Slobodova, Sarka Zehnalova, Milos Kudelka, Petra Schneiderova, Regina Fillerova, Jiri Gallo

**Affiliations:** 1 Department of Immunology, Faculty of Medicine and Dentistry, Palacky University Olomouc and University Hospital Olomouc, Olomouc, Czech Republic; 2 Department of Clinical and Molecular Pathology, Faculty of Medicine and Dentistry, Palacky University Olomouc and University Hospital Olomouc, Olomouc, Czech Republic; 3 Department of Computer Science, Faculty of Electrical Engineering and Computer Science, VSB-Technical University of Ostrava, Ostrava, Czech Republic; 4 Department of Orthopaedics, Faculty of Medicine and Dentistry, Palacky University Olomouc and University Hospital Olomouc, Olomouc, Czech Republic; University of Chile, CHILE

## Abstract

**Objective:**

Aseptic loosening (AL) is the most frequent long-term reason for revision of total knee arthroplasty (TKA) affecting about 15–20% patients within 20 years after the surgery. Although there is a solid body of evidence about the crucial role of inflammation in the AL pathogenesis, scared information on inflammation signature and its time-axis in tissues around TKA exists.

**Design:**

The inflammation protein signatures in pseudosynovial tissues collected at revision surgery from patients with AL (AL, n = 12) and those with no clinical/radiographic signs of AL (non-AL, n = 9) were investigated by Proximity Extension Assay (PEA)-Immunoassay and immunohistochemistry.

**Results:**

AL tissues had elevated levels of TNF-family members sTNFR2, TNFSF14, sFasL, sBAFF, cytokines/chemokines IL8, CCL2, IL1RA/IL36, sIL6R, and growth factors sAREG, CSF1, comparing to non-AL. High interindividual variability in protein levels was evident particularly in non-AL. Levels of sTNFR2, sBAFF, IL8, sIL6R, and MPO discriminated between AL and non-AL and were associated with the time from index surgery, suggesting the cumulative character of inflammatory osteolytic response to prosthetic byproducts. The source of elevated inflammatory molecules was macrophages and multinucleated osteoclast-like cells in AL and histiocytes and osteoclast-like cells in non-AL tissues, respectively. All proteins were present in higher levels in osteoclast-like cells than in macrophages.

**Conclusions:**

Our study revealed a differential inflammation signature between AL and non-AL stages of TKA. It also highlighted the unique patient’s response to TKA in non-AL stages. Further confirmation of our preliminary results on a larger cohort is needed. Analysis of the time-axis of processes ongoing around TKA implantation may help to understand the mechanisms driving periprosthetic bone resorption needed for diagnostic/preventative strategies.

## Introduction

Total knee arthroplasty (TKA) is one of the most effective therapies of end-stage osteoarthritis with growing numbers of patients operated each year worldwide [1]. Currently, the total number is estimated at about 3 million cases per year. Aseptic loosening (AL) is the most common late failure of TKA with estimated occurrence about 15–20% of patients in a 20-year time horizon [2]. It is always accompanied with periprosthetic osteolysis (PPOL), which is a scientific synonym for clinically observed bone defects. These complicate the reoperation of aseptically loosened TKA, increase its cost, and limit survivorship of the revision TKA. The knowledge on the pathogenesis of AL is, therefore, crucial to develop effective preventative strategies. The molecular mechanisms underlying PPOL/AL in TKA are poorly understood. Some researchers emphasize the role of tissue inflammation stimulated in the response to prosthetic byproducts by innate immunity network similarly like in total hip arthroplasty (THA) [3, 4].

Contemporary biological theory links resident tissue cells [5] to maintaining local tissue homeostasis around TKA contributing to stable and functional TKA for many years [6]. However, the continuous burden of wear particles, liberating from the articulation surfaces of TKA during each step and migrating around bone-implant interface [7], induce release of pro-inflammatory mediators triggering and perpetuating chronic low-grade inflammation around TKA [8]. This chronic inflammatory condition favours secretion of a number of osteoclastogenic mediators, namely the receptor activator of nuclear factor kappa-B (RANK) ligand and others, which in turn promote differentiation of macrophages into osteoclasts and multinucleated giant cells, effecting bone resorption [9–11]. In addition, tissue ischemia, cell necrosis [12, 13], an increased amount of joint fluid [14], all resulting from chronic particle/ion load, and synovitis can contribute to non-resolved inflammation and PPOL leading eventually to AL [6]. TKA also faces continual mechanical load inducing chronic stress and strain imbalances at the bone-implant interface that could contribute to trabecular bone resorption leading potentially to a loss of mechanical fixation [15].

Although the proteomic analysis of tissues around the TKA may lead to a better understanding of the complex interaction between the prosthetic device and its surrounding tissue, the proteomic tissue signature related to PPOL/AL is poorly elucidated [16]. Only one proteomic study on 29 inflammatory molecules in tissues from aseptically loosened hips has thus far been reported [17], and no study about loosened knees is available. The current knowledge comes from several emerging studies on serum and/or synovial fluid proteins from late PPOL/AL [18–22]. Importantly, no information related to conditions predating PPOL/AL is available.

In this pilot study, we attempted to examine the inflammation signature in tissues taken in patients with TKA in AL (covering also PPOL) and non-AL stages, with special emphasis on the TKA lifetime. The results obtained by ultra-sensitive and specific PEA Immunoassay were confirmed by immunohistochemistry. We believe that characterisation of particular phases of AL is of utmost importance for a better understanding of the underlying pathomechanisms and development of diagnostic/preventative/therapeutic strategies for improvement of care on patients undergoing TKA.

## Material and methods

### Subjects

**Fig 1:**
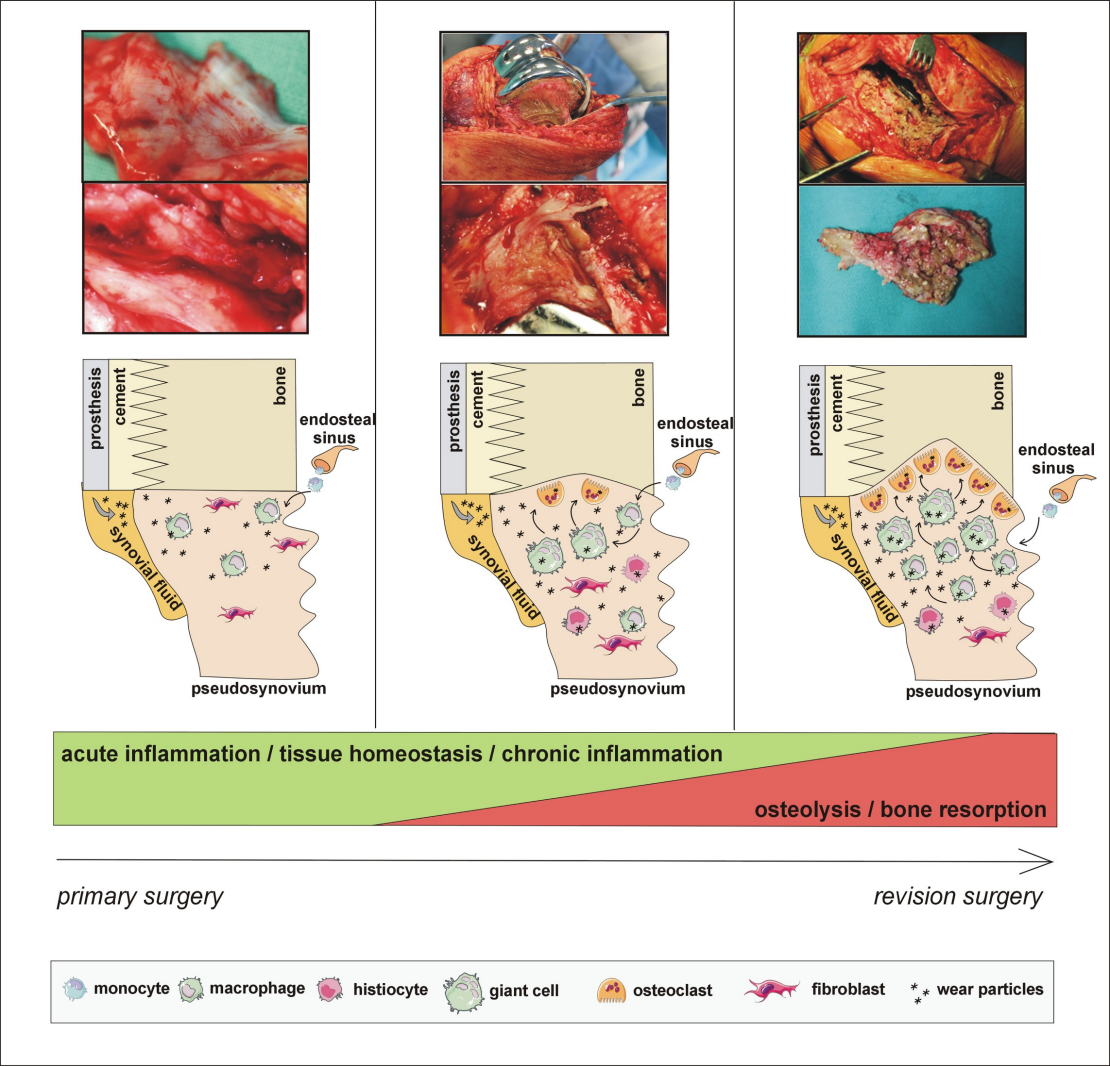
Representative samples of pseudosynovial tissues retrieved during the revision surgery. The proposed concept of events undergoing in the periprosthetic tissues is associated with a gradual loss of local tissue homeostasis in favour of low-grade inflammatory changes with growing predominance of osteoclasts at the implant-bone interface leading eventually to periprosthetic bone resorption (PPOL). **Left panel:** an early thin tissue retrieved from a stable TKA without signs of PPOL; **Middle panel:** a pseudosynovial tissue covering the inner space of stable TKA without PPOL; **Right panel:** a well-developed pseudosynovial membrane in aseptically loosened TKA.

Pseudosynovial tissues (Fig 1) were obtained from TKA patients re-operated for AL (AL, n = 12) and non-AL patients (non-AL, n = 9) re-operated for other reasons (pain of unknown origin, instability, fracture) than AL, with no clinical and radiographic signs of PPOL/AL at the time of sampling. All patients were enrolled in a single tertiary center between 2009 and 2014. Stability of the TKA was examined intra-operatively by the experienced surgeon harvesting periprosthetic tissues. For detailed patient characteristics, see Table 1. Infection cases were excluded according to the previously published diagnostic algorithm [23].

**Table 1:** Clinical characteristics of enrolled patients with TKA; all reoperated TKAs were cruciate retaining implants with fixed bearing polyethylene surfaces.

	Non-AL	AL
**Patients**, N	9	12
**Gender** (male/female)	4/5	3/9
**BMI** [kg/m^2^]	30.1	29.9
(median, min-max)	(22.9–38.9)	(22.9–36.1)
**Primary diagnosis**		
Primary OA	7	12
Posttraumatic arthritis	2	0
**Age** [yrs] (median, min-max)		
**at primary surgery**^&^	63 (56–83)	65 (54–74)
**at revision surgery**^§^	66 (60–83)	78 (67–84)
**Time from index to revision surgery** [months]	41	138
(median, min-max)	(2–123)	(36–202)
**Reason for revision surgery:**		
Pain, stable TKA, firmly fixed implant	4	0
Unstable TKA, firmly fixed implant	2	0
Periprosthetic fracture, firmly fixed implant	3	0
Aseptic loosening of TKA	0	12
**Type of fixation:**		
Cemented	9	11
Partially cemented	0	1
**KSS pain before revision surgery**	62.2	43.2
(median, min-max)	(40–80)	(35–63)
**KSS function before revision surgery**	60.3	47.1
(median, min-max)	(20–75)	(40–60)
**Size of femoral bone defects:**		
F1	9	8
F2A	0	2
F2B	0	1
F3	0	1
**Size of tibial bone defects:**		
T1	7	7
T2A	2	5
**Polyethylene damage:**		
None	5	1
Small	3	3
Intermediate	0	2
Severe	1	6
**Synovial fluid CRP at revision surgery [mg/L]:**		
<5	4	11
5–10	3	0
>10	2	1

^&^*P* = 0.374

^§^*P* = 0.006

Written informed consent about the use of periprosthetic tissues for the purpose of this study was obtained from each subject prior to inclusion in the study. The Ethical Committee for the Faculty of Medicine and Dentistry, Palacky University Olomouc and the University Hospital Olomouc approved this study in accordance with the Helsinki Declaration (MZ ČR VES16-31852A).

### Periprosthetic tissue sample processing, protein concentration assessment

Tissue specimens (a thin surface layer of tissue covering the inner side of a joint capsule) approximately 2x5 mm in diameter were taken from a representative and readily accessible area of the joint capsule and evaluated macroscopically by a single surgeon. Tissue samples were taken and processed as described below. There is already evidence that this tissue carries relevant information on the host response to wear particles, as it is in permanent contact with the prosthetic byproducts, signals of danger/tissue damage all dispersed in joint fluid, and it has been as well under repetitive pressure of joint fluid until the time of the revision surgery [24, 25].

For the preparation of tissue lysates, tissues removed from RNAlater were homogenised in a cold RIPA buffer (Sigma Aldrich, St. Louis, USA) containing the Protease inhibitor cocktail (Sigma Aldrich, St. Louis, USA; 1:100) using a tissue homogeniser (ULTRA-TURRAX Homogeniser, Sigma Aldrich) on ice. After measurement of protein concentrations with a Pierce BCA Protein Assay Kit (Thermo Scientific, Rockford, IL), cell lysates were diluted to the same concentrations (1mg/ml) using a cold RIPA buffer containing the Protease inhibitor cocktail (1:100) and stored at -80°C until use [24].

### Antibody-based Proximity Extension Assay (PEA)

The protein levels of molecules associated with inflammation and osteoclastogenesis (see S1 Table) were assessed on a 92-plex Targeted Proteomics Chip (Proseek Multiplex Oncology I kit, Olink Bioscience, Uppsala, Sweden) using 1 μl of tissue lysates as reported previously [26, 27]. For sensitivity and specificity parameters, see Assarsson et al. [28].

### Histopathology and immunohistochemical analysis

For histopathological evaluation, the periprosthetic tissues consisting of only neo-synovium, but not bone cavities, were fixed in 10% buffered formalin and embedded in paraffin [24]. One single specimen was available per case. The paraffin sections were stained using a combination of hematoxylin and eosin (H&E) dyes as well as immunohistochemistry for AREG, TNFR2, CCL2, IL8, and TRAP as previously described [29]. An immunohistoscore, antibodies and their dilutions used in this study see S1 Text.

### Multivariate patient similarity networks

The method of network (graph) construction based on the nearest neighbour analysis [30, 31] was applied for i) identification of the most discriminant proteins between AL and non-AL and ii) visualisation of individual patients within similarity networks based on the similarities in tissue protein levels. For the identification of key markers distinguishing the particular diagnostic subgroups, a co-occurrence network was formed. In this network, markers are vertices and co-occurrences with their nearest neighbours are edges. As input for co-occurrence analysis, the most discriminant combinations of three markers resulting from MDA (multilinear discriminant analysis) were used. The internal structure of the patient network represents the similarity of the immunophenotypic profiles among the patients. The nearest neighbours in the network are linked by edges and have the highest similarity in terms of MFI expression levels or the positivity/negativity of the investigated markers. In the visualised patient similarity network, colours distinguish the particular diagnostic subgroups. Weighted network modularity [32] and silhouettes were used to assess the quality of the dividing of each patient into the particular subgroup.

### Data analysis

Statistical analyses (Mann-Whitney test, Benjamini-Hochberg correction, Spearman correlations) were performed using R statistical software with the Caret package (http://www.r-project.org/; http://topepo.github.io/caret/index.html). Data analyses were performed on linearised expression data (2ddCq). *P*-values were adjusted for multiple comparisons using the False Discovery Rate according to the Benjamini-Hochberg procedure; *P*_*corr*_ value < 0.05 was considered as significant.

## Results

### Tissue protein pattern associated with AL/non-AL of TKA

**Fig 2:**
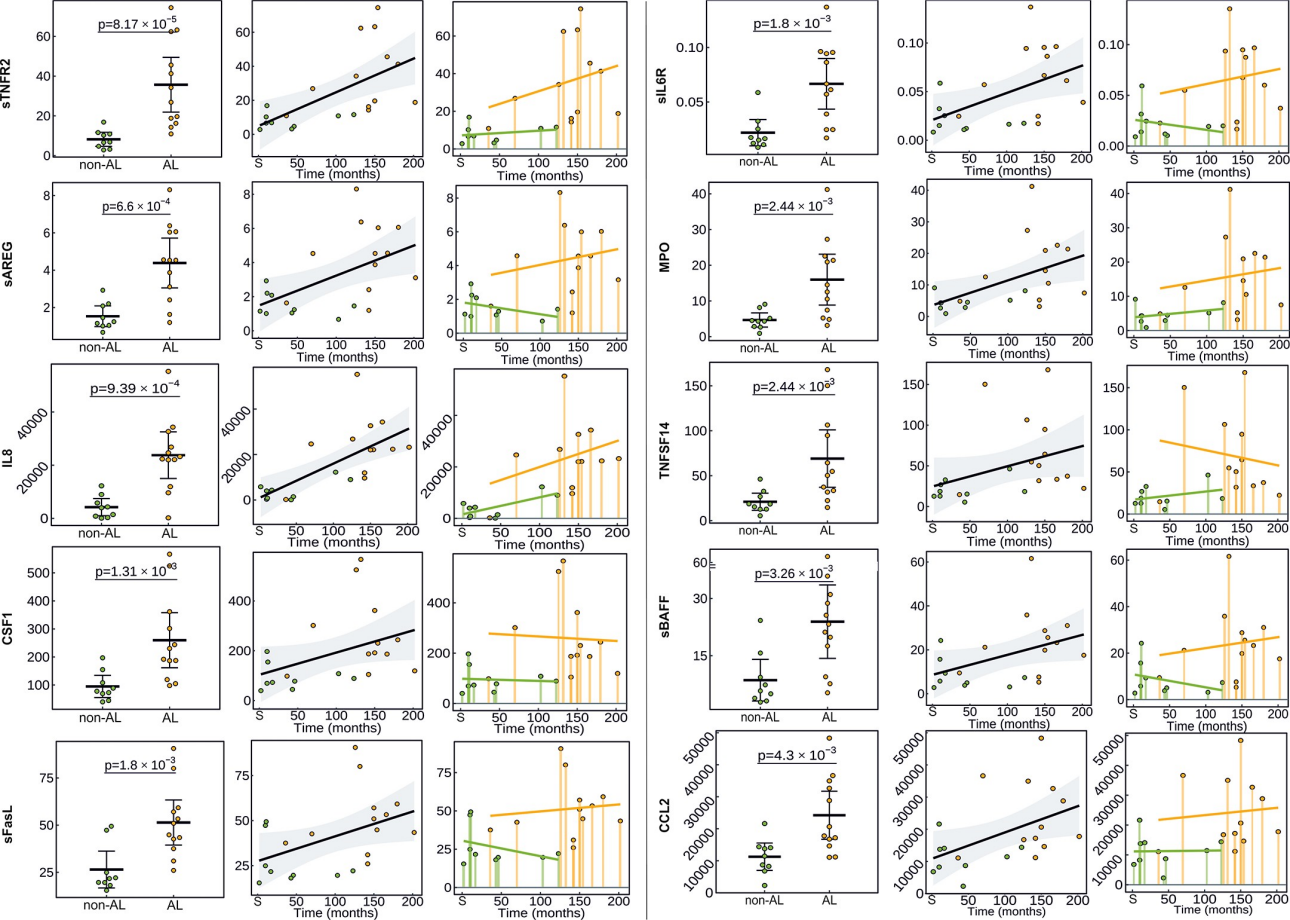
Protein levels of top-deregulated proteins differentially expressed in tissues from TKA patients. Protein levels of top-deregulated proteins in pseudosynovial membrane lysates from patients with aseptic loosening (AL, yellow dots/columns) and non-aseptic loosening (non-AL, green dots/columns) stages (left panel) and their relationship with implant lifetime (middle/right panel) are presented. The y-axis represents the normalized protein expression. The x-axis represents the implant lifetime in months from primary TKA surgery (S). Horizontal bars indicate group means, and diagonal bars indicate the trend of protein levels over time; error bars indicate 95% confidence interval.

Ninety-two proteins were assessed in lysates from tissues sampled during AL and non-AL reoperations in TKA patients. When comparing AL to non-AL, thirteen analytes (sTNFR2, sAREG, IL8, CSF1, sFasL, sIL6R, MPO, TNFSF14, sBAFF, CCL2, KLK6, IL1RA, suPAR) were upregulated in AL (P_*corr*_<0.05, Table 2, Fig 2) and additional twenty proteins were deregulated (S1 Fig, S2 Table), but the differences did not reach significance after multiple comparison adjustment. The levels of 49 proteins did not differ between subgroups (S2 Table) and the levels of CA125, CA242, sCAIX, CEA, EPO, sER, KLK11, MIA, MMP3, PSA were below the detection limit and thus excluded from further analysis.

**Table 2:** Levels of proteins in tissue lysates found deregulated between TKA patients with/without aseptic loosening (AL / non-AL).

*Analyte*	*Mean Linear ddCq (95% CI)*		*FC*	*P*	*P*_*corr*_
	*Non-AL*	*AL*			
sTNFR2	8.2 (4.7–11.7)	35.7 (21.9–49.5)	4.47	8.165 × 10^−5^	6.70 × 10^−3^
sAREG	1.5 (1.0–2.1)	4.4 (3.1–5.7)	3.65	6.600 × 10^−4^	2.46 × 10^−2^
IL-8/CXCL8	4,225 (997–7,453)	23,758 (14,991–32,524)	5.68	9.390 × 10^−4^	2.46 × 10^−2^
CSF1	94.8 (55.2–134.4)	259.7 (161.5–357.9)	2.71	1.306 × 10^−3^	2.46 × 10^−2^
sFasL	26.5 (16.7–36.2)	51.4 (39.4–63.3)	2.21	1.803 × 10^−3^	2.46 × 10^−2^
sIL-6R	0.022 (0.010–0.034)	0.067 (0.043–0.090)	3.89	1.803 × 10^−3^	2.46 × 10^−2^
MPO	4.7 (2.7–6.7)	16.0 (8.9–23.1)	3.12	2.436 × 10^−3^	2.50 × 10^−2^
TNFSF14	21.0 (11.5–30.6)	69.1 (37.1–101.0)	2.84	2.436 × 10^−3^	2.50 × 10^−2^
sBAFF	8.6 (3.1–14.0)	23.9 (14.3–33.5)	3.85	3.260 × 10^−3^	2.97 × 10^−2^
CCL2/MCP1	11,257 (6,979–15,535)	24,218 (16,709–31,728)	1.68	4.300 × 10^−3^	3.53 × 10^−2^
KLK6	4.2 (2.2–6.2)	9.8 (6.6–12.9)	3.33	5.620 × 10^−3^	4.19 × 10^−2^
IL1-RA	2,489 (1,736–3,242)	4,519 (3,306–5,732)	1.68	7.253 × 10^−3^	4.58 × 10^−2^
suPAR	40.0 (26.4–53.5)	68.4 (51.5–85.3)	2.09	7.253 × 10^−3^	4.58 × 10^−2^
IFNγ	0.038 (0.027–0.050)	0.070 (0.047–0.093)	1.95	0.012	6.43 × 10^−2^
TRAP	1,1367 (577–1,696)	2,431 (1,606–3,256)	2.14	0.012	6.43 × 10^−2^
sCD30L	33.0 (20.2–45.8)	57.8 (42.1–73.6)	2.26	0.015	6.73 × 10^−2^
sIL17RB	0.031 (0.017–0.045)	0.062 (0.041–0.083)	2.68	0.015	6.73 × 10^−2^
MYD88	8.5 (4.1–12.9)	13.4 (9.9–16.9)	2.17	0.015	6.73 × 10^−2^
sCD69	113.9 (40.3–187.6)	240.0 (140.4–339.6)	2.78	0.023	8.87 × 10^−2^
IL6	272.4 (61.6–483.1)	1,242 (375–2,109)	3.89	0.023	8.87 × 10^−2^
sTNFR1	82.7 (36.2–129.2)	139.7 (104.4–175.1)	2.21	0.023	8.87 × 10^−2^
CXCL10	2,151 (-6,967–4,999)	2,909 (1,785–4,033)	2.67	0.028	9.51 × 10^−2^
sMICA	52.3 (19.6–85.1)	103.2 (63.4–143.0)	2.18	0.028	9.51 × 10^−2^
PRL	1.4 (0.7–2.2)	3.0 (1.9–4.2)	3.07	0.028	9.51 × 10^−2^
sBTC	249.3 (136.8–361.8)	394.7 (281.2–508.1)	2.01	0.034	0.100
sTGFA	5.3 (1.5–9.1)	8.1 (5.8–10.5)	2.20	0.034	0.100
sTNFRSF4	2.7 (1.4–3.9)	4.3 (3.0–5.6)	1.87	0.034	0.100
CXCL9	59.4 (-7.7–126.4)	125.3 (42.1–208.4)	3.35	0.041	0.100
sE selectin	0.8 (0.3–1.2)	1.7 (0.9–2.4)	3.11	0.041	0.100
Galectin 3	16.2 (8.1–24.4)	29.9 (19.4–40.5)	2.65	0.041	0.100
PRSS8	1.3 (0.6–2.0)	2.5 (1.4–3.6)	2.04	0.041	0.100
THPO	0.087 (0.047–0.100)	0.200 (0.100–0.300)	2.50	0.041	0.100
TGFB1	15.7 (10.0–21.5)	28.4 (17.9–38.8)	1.52	0.049	0.120

FC: fold change

All the data are presented as Mean Expression Level (95% CI).

*P*corr: value corrected for multiple comparisons (Benjamini-Hochberg correction)

### Correlation of protein levels with the implant lifetime

When the levels of proteins in relation to the implant lifetime were assessed, higher levels of four TNF-family members (sTNFR2, TNFSF14, sFasL, sBAFF), cytokines/chemokines (IL8, IL1RA, sIL6R, sIL17RB, IFNγ, CCL2, CCL19), growth factors (sAREG, CSF1), and others (TRAP, sCD69, MPO, sPECAM1, suPAR, Galectin 3, KLK6) were associated with longer time between primary and revision surgery (*P*<0.05, S3 Table, Fig 2). Only chemokine CCL21 levels decreased over time (*P*<0.05, S3 Table, S1 Fig).

In non-AL, five growth factors/angiogenic proteins (sTIE2, sVEGFR2, PGF, sHGF, sE-selectin) correlated with the time to revision surgery (*P*<0.05, S3 Table, S2 Fig). In AL, only CXCL10 levels correlated with the longer implant life (*P*<0.05, S3 Table, S2 Fig).

### Histopathological analysis of AL and non-AL tissues

**Fig 3:**
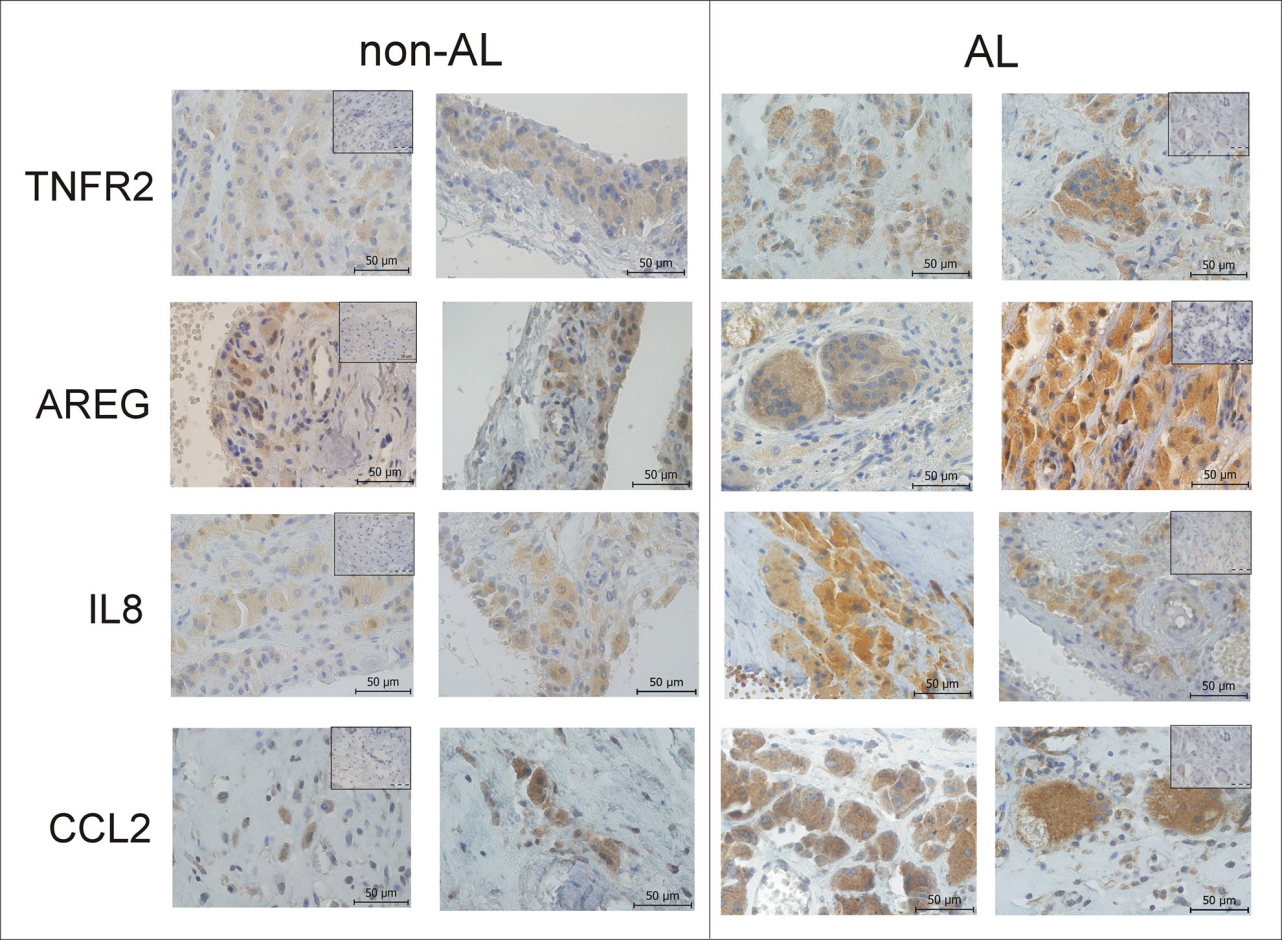
Immunohistochemical staining for TNFR2, AREG, IL8, and CCL2 in pseudosynovial membrane sections of TKA patients. Pseudosynovial tissues were obtained from patients reoperated for aseptic loosening (AL) and non-aseptic loosening (non-AL)–representative examples. Stainings with a non-specific isotype control antibody are shown in the upper segments of the panels. Scale bar: 50 μm.

Histopathological analysis of AL and non-AL tissues showed high interindividual variability and differences in the amount and type of cellular infiltration, with the highest variability in tissues taken during non-AL stages. Non-AL tissues showed the low intensity of synovitis, which was milder in the early period (P1, P2, P3) and increased with longer time of implant service (P4) (Fig 3). It was characterised by a low number of macrophages and sporadic lymphocytes spread in fibrous tissue below the synovial surface containing an increased amount of synovialocytes. Vascularity was only slightly increased and occasionally present multinucleated giant cells with a low number of nuclei were found under the neo-synovial surface. These cells did not contain wear particles. Wear particles were present inside of multinucleated giant cells only in samples with the longest time from initial surgery (P4) together with particle laden macrophages. H&E staining focused on the neo-synovial surface in cases, when the synovial layer was retained. In samples without neo-synovial surface, the closest representative area was captured.

In AL tissues, high intensity of synovitis was present. The neo-synovial surface was damaged or covered by a necrotic layer. Below this area, increased infiltrate of macrophages and multinucleated giant cells with a high number of nuclei were predominantly detected. Lymphocytic infiltrate was minimal only in perivascular localisation. Besides particle laden macrophages containing particles, irregularly distributed multinucleated giant cells contained fibrilar polarized material present in all AL cases (Fig 3).

The staining with TRAP confirmed the presence of a low number of multinucleated osteoclast-like giant cells with only a few nuclei in non-AL (Fig 3) with an increasing presence of multinucleated osteoclast-like cells with a higher number of nuclei in patients with a longer implant lifetime (Fig 3). AL tissues were characterised by increased accumulation of giant osteoclast-like cells with a high number of nuclei (Fig 3). TNFR2, AREG, IL8, and CCL2 staining was positive in both AL and non-AL tissues identifying these proteins in macrophages and multinucleated osteoclast-like cells.

### Immunohistochemical analysis of non-AL and AL tissues around TKA

**Fig 4:**
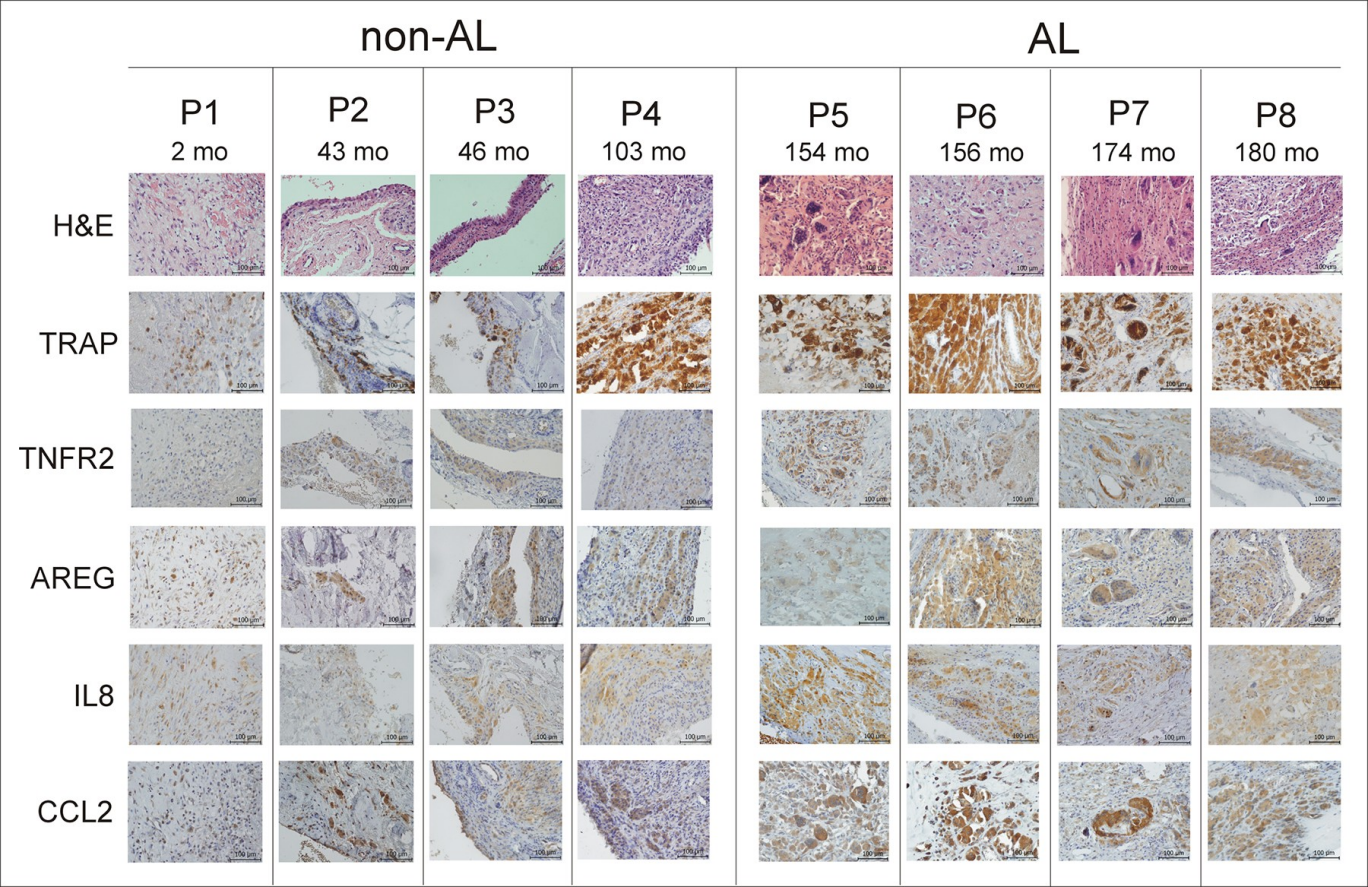
H&E and immunohistochemical staining for TRAP, TNFR2, AREG, IL8 and CCL2 in AL and non-AL tissues of TKA patients. Pseudosynovial membrane sections obtained from TKA patients with the stable implant and no signs of aseptic loosening (non-AL, P1-P4) and those with aseptic loosening (AL, P5-P8). Months (mo) indicate the time between primary and revision surgery. H&E staining shows neo-synovial surface when present, otherwise the area below was captured. Immunohistochemical staining shows representative areas with highest cell positivity, focused on macrophages and multinucleated osteoclast-like giant cells. Scale bar: 100 μm.

**Fig 5:**
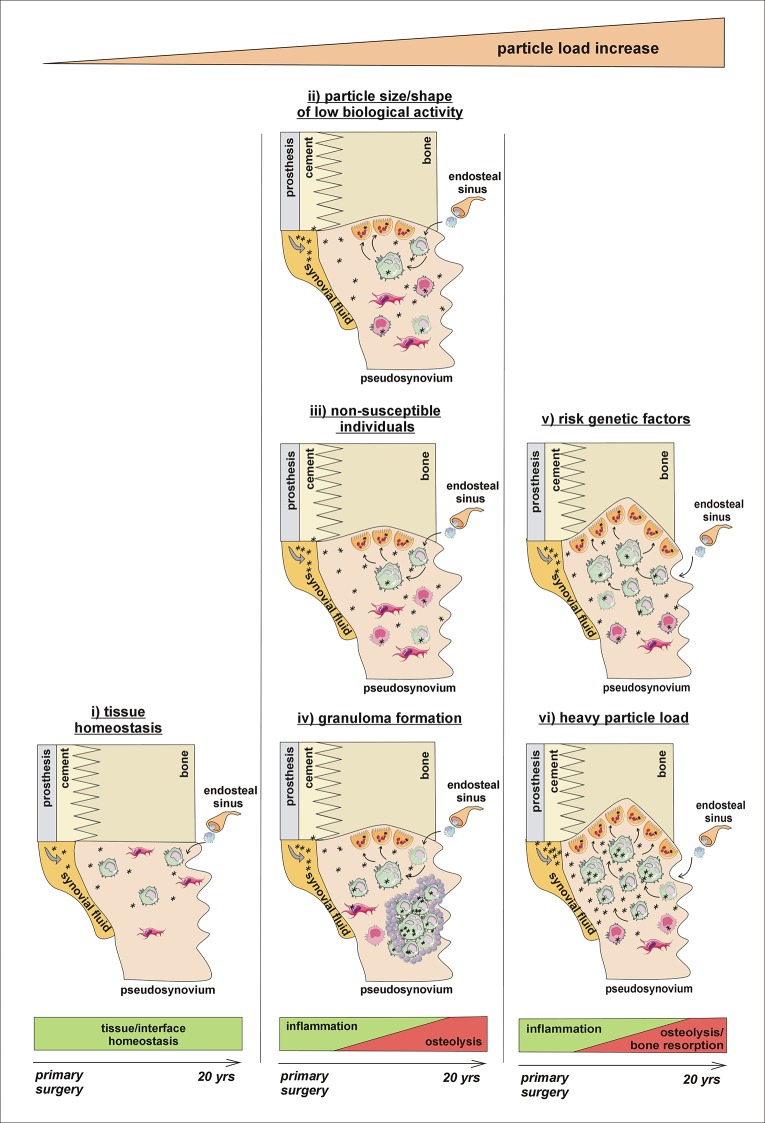
Proposed major scenarios in response to wear particles liberated into the implant surroundings. Low particle load provokes an inflammatory response and activation of macrophages and other immune cells, which eliminate foreign-body particles, thus enabling local tissue homeostasis (left panel) (**i**). Higher particle load stimulates formation of multinucleated giant cells, differentiating in response to inflammatory/osteoclastogenic factors into osteoclasts and accumulating at the bone-implant interface; however, in the case of (**ii**) particle size/shape of low biological activity, (**iii**) non-susceptible individuals with protective genetic/individual parameters, or (**iv**) individuals with encapsulation of wear particles into granulomas, low-grade chronic inflammation predominates over osteoresorptive processes (middle panel). On the contrary, the bone resorption predominates over inflammation in (**v**) individuals with genetic/ individual parameters predisposing to osteolysis and in (**vi**) those individuals with heavy particle load, where the formation of pseudosynovium with a high number of osteoclasts, executing bone resorption, is observed at the bone-implant interface (right panel). Our theory does not exclude multiple other factors (e.g. fluid pressure, mechanically induced bone resorption, interference of TKA body/fixation interface with the biomechanical characteristics of the host tissue, quality of the host tissue, local and general reactivity) that may also contribute to implant failure.

Top-deregulated proteins TNFR2, AREG, IL8, CCL2 and TRAP were assessed in tissue biopsies using immunohistochemistry. As the distribution of positive cells was uneven, the area with high cell positivity was taken. Overall, in AL tissues, giant osteoclast-like cells with a high number of nuclei primarily produced the studied proteins, whereas, in non-AL, the proteins have primarily been localised to histiocytes and osteoclast-like cells with only a few nuclei (Fig 4). All proteins were present in higher levels in multinucleated osteoclast-like cells than in macrophages (Fig 4). When comparing AL and non-AL, trend to higher TNFR2, AREG, IL8, and CCL2 levels was found in macrophages and multinucleated osteoclast-like cells in AL as assessed by semiquantitative analysis. The proposed major scenarios of processes ongoing in tissues around TKA in response to wear particles liberated into the implant surroundings shows Fig 5.

### Multivariate patient similarity networks

**Fig 6:**
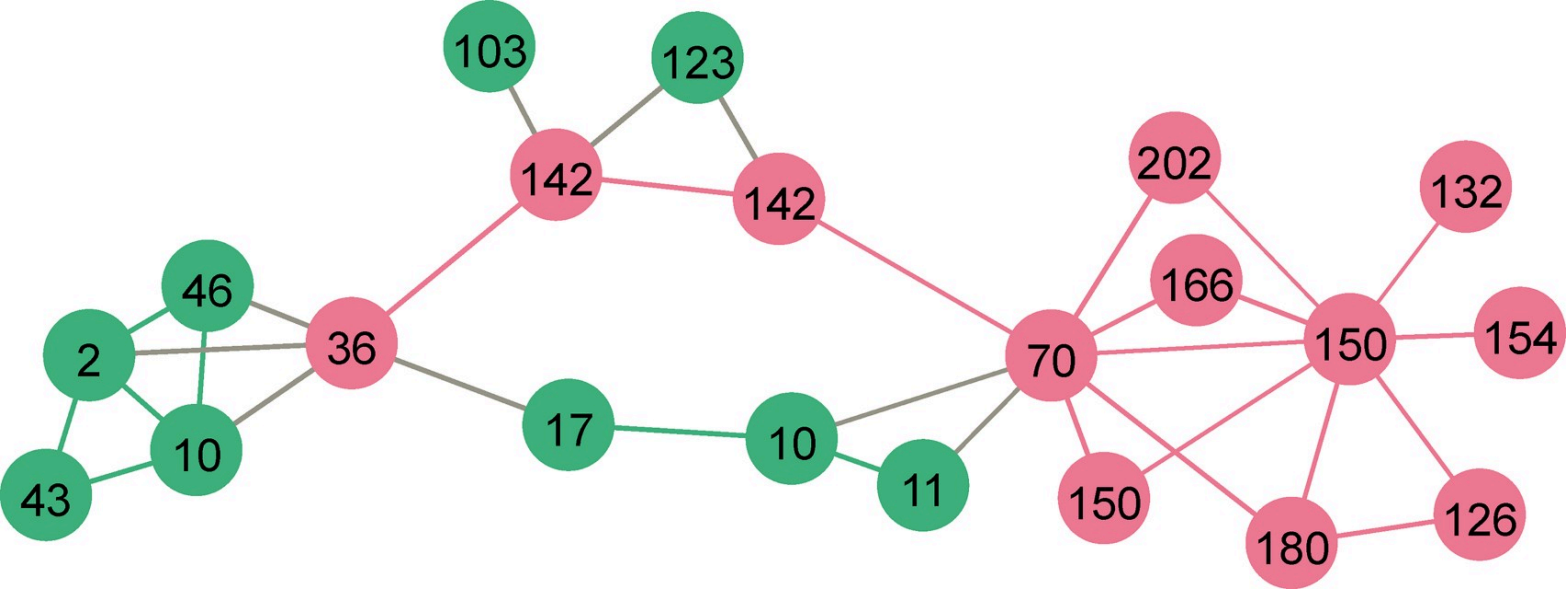
Patient similarity network of the most discriminant proteins in tissue lysates from TKA patients. Patient similarity network was constructed based on the similarity in protein levels (sAREG, sBAFF, IL8, sIL6R, MPO, and sTNFR2) in tissues around TKA from aseptic loosening (AL, red vertices) and non-aseptic loosening (non-AL, green vertices). The number in each vertex (= patient) indicates the implant lifetime (time from primary to revision surgery) for each particular patient in months.

To identify the most informative proteins distinguishing between AL and non-AL, the multivariate patient similarity networks based on different combinations of proteins were constructed and modularity values were measured. A combination with a small number of proteins with high modularity revealed sAREG, sBAFF, IL8, sIL6R, MPO, and sTNFR2 as the most discriminant for AL (Fig 6). AL patients with a lengthy time between primary and revision surgery formed one distinct cluster. Non-AL patients with a short time to the revision surgery formed another distinct cluster (Fig 6). There were two patient’s groups (= clusters) in-between clustering together based on a similarity in protein levels, including both AL and non-AL: the first with a long, and the second with a short implant lifetime (Fig 6).

## Discussion

Although there is a growing body of evidence about the crucial role of inflammation in AL, only a single proteomic study in tissues of thirteen patients from aseptically failed hips investigating 29 inflammatory molecules [17] exists. No information is relevant to non-AL stages and aseptically failed TKA. We therefore attempted to investigate inflammatory molecules in tissues in aseptically failed TKA as well as unique stages predating AL (non-AL) using highly sensitive PEA Immunoassay. When comparing AL to non-AL, TNF-family members (sTNFR2, TNFSF14, sFasL, and sBAFF), cytokines/chemokines (IL8, CCL2, IL1RA/IL36, sIL6R), and growth factors (sAREG and CSF1) were present at higher levels in AL. The most up-regulated sTNFR2 in AL was already reported as a key molecule for osteoclast formation, and absence of both receptor subtypes (TNFR1/TNFR2) virtually abrogated osteoclastogenesis [33]. The importance of TNF receptors for osteoclast formation induced by TNF-α was proven in mice deficient in these receptors [34]. Also other TNF family members (TNFSF14, FasL, and BAFF) have been found essentially involved in osteoclastogenesis: LIGHT/TNFSF14 increased osteoclastogenesis in multiple myeloma-bone disease [35], FasL enhanced the osteoclasts differentiation [36] and sBAFF contributed to osteoclast differentiation [37] through activation of an alternative NF-κB pathway [38].

IL8 and CCL2 were among the up-regulated chemokines in AL. Elevated IL8 has been already found in aseptically failed tissues in THA [17], reaffirming its prominent role in osteoblast-mediated osteoclastogenesis and end-stage bone resorption [17, 39]. IL8 also directly stimulates the differentiation of human peripheral blood mononuclear cells into bone-resorbing osteoclasts [40]. Importantly, IL8 stimulates osteolysis independently of the RANKL pathway [41]. However, IL8 connection with the macrophage proliferation and their bone invasion is not clear and deserves future investigation. CCL2 is another key chemokine essential for the formation of osteoclasts and foreign body giant cells [42]. Its importance is supported by the evidence of reduced wear particle-induced osteolysis and inflammation *in vivo* after the delivery of mutant CCL2 protein [43, 44].

Our study also revealed the up-regulation of sIL6R, but only a slight elevation of IL6, in AL. There is current evidence that the IL6/sIL6R system regulates RANKL-induced osteoclast formation *via* specifically modulating phosphorylation of NF-κB, ERK, and JNK in a RANKL concentration-dependent manner [45]. Our observation of low IL6 in AL is in line with a previous report where IL6 was not indicative of osteolysis and AL [17]. The next elevated protein was IL1RA, a competitive receptor antagonist able to block the binding of IL1α and IL1β to IL1RI, thereby preventing IL1RI activation and inhibiting the biological action of pro-inflammatory and pro-osteoclastogenic IL1 [46, 47]. Also CSF1, a critical growth factor for macrophage development was shown to induce the key osteoclastogenic receptor RANK in osteoclast precursors, thus mediating TNF-induced inflammatory osteolysis in mouse models [48]. Regarding myeloperoxidase (MPO), the elevated levels may result from innate immune infiltrates at the sites of inflammation and injury around TKA. However, a recent study reported its involvement in the inhibition of osteoclast differentiation and bone resorption [49] thus further studies are needed to clarify its role in AL.

A release of membrane-bound epidermal growth factor (EGF)-like growth factors, including amphiregulin (AREG), HB-EGF, and TGF-α have been shown to suppress the expression of osteoprotegerin (OPG) in osteoblasts and subsequently, potentiate osteoclast differentiation as shown in bone metastasis [50]. Interestingly, sAREG was highly elevated in AL tissues. There is evidence that this signalling molecule, contained in exosomes, is able to induce osteoclast differentiation through the activation of the EGFR pathway in pre-osteoclasts that in turn causes increased expression of RANKL [51]. The importance of AREG was also evident from patient similarity networks, where sAREG together with sBAFF, IL8, sIL6R, MPO, and sTNFR2 discriminated between AL and non-AL tissues. Interestingly, four distinct patient groups were formed based on the similarity of the protein levels: one group with only AL and one with non-AL patients, and two in-between groups showing similarity in implant lifetime regardless of the presence of AL. This observation furthermore supports a time-dependent manner of inflammatory and bone-resorptive processes around TKA, thus deserving future investigations.

Next, our proteomic analysis revealed a high variability of levels of studied proteins in non-AL tissues. This fact suggests the patient’s unique response to TKA after implantation. This could be explained in the context of variability in the amount of released wear particles and their clearance by immune cells in individual patients. The levels of inflammatory/adhesion molecules sTIE2, sVEGFR2, PGF, sHGF, and sE-selectin in non-AL tissues taken early after reoperations were higher than those with longer prosthesis lifetime, thus supporting the hypothesis that angiogenic/remodelling processes are activated early after surgery. A low amount of histiocytes and occasionally present small osteoclast-like cells were detected in non-AL tissues, which were identified as the main source of pro-osteoclastogenic molecules. Generally, low levels of pro-osteoclastogenic proteins were detected in non-AL.

In AL, the majority of elevated proteins have been associated with osteoclastogenesis and the formation of giant multinucleated cells in previous studies. Tissues from AL showed higher macrophage infiltration and the presence of multinucleated giant osteoclast-like cells, as shown by staining with TRAP, a marker of osteoclasts. Interestingly, those cells accumulating wear particles were found as the main producers of pro-osteoclastogenic proteins in this study. The levels of elevated proteins were associated with implant lifetime, suggesting the cumulative character of pro-osteoclastogenic response to prosthetic byproducts. In line with observation in loosened THA [17], our data revealed an association of chemokine CXCL10 with implant lifetime in TKA, assuming a key role of T-cells in AL. AL tissues also showed high interindividual heterogeneity in protein levels, which may result from differences in particle load and particle size/shape [52, 53], the ability of the immune system to resolve chronic inflammation and individual genetic susceptibility to PPOL/AL [54, 55]. Moreover, the control of the “deposition” of wear particles via their disposal in tissue further from the implant [56] could also co-influence the survivorship of TKA. Taken together, our data further confirmed the key role of pro-osteoclastogenic players to AL, the need to investigate the time-axis of their production, as well as highlights the possibility to target them as shown already for CCL2 [43, 44].

This study has several limitations. In addition to the studied molecules from 92-plex targeted proteomics chip, other mediators/pathways may also be involved in the development of AL. Moreover, there is a partial overlap in terminology between AL and PPOL: AL is always accompanied by PPOL in clinical practice, however, PPOL can be identified in some patients without AL. Thus, we selected a unique cohort of non-AL patients with no sign of PPOL to obtain relevant results. We believe that our preliminary study, even on a small-size patient cohort, provides the first tracks to better understand the expression of molecules on established time points and shows the need to analyse the time-dependent axis of processes ongoing around TKA in future studies.

## Conclusions

This first proteomic analysis of the inflammation time-axis in TKA tissues revealed the cumulative nature of the inflammatory response to prosthetic byproducts. The majority of proteins elevated in AL were associated with osteoclastogenesis and formation of giant multinucleated cells, whereas many of them were not linked to AL previously. Levels of inflammatory molecules were associated with the time elapsed from the index surgery, thus suggesting the cumulative character of tissue changes induced and perpetuated in response to prosthetic byproducts. The understanding of processes predating AL and better characterisation of the time-axis of inflammatory and osteoresorptive processes may help to propose effective preventative/therapeutic strategies contributing to the prolongation of the prosthesis lifetime. The results of our preliminary study need to be confirmed in a larger patient cohort.

## Supporting information

S1 Table List of investigated proteins. (DOCX)

S2 Table Protein levels in tissue lysates from TKA patients with/without aseptic loosening (AL / non-AL) of TKA. (DOCX)

S3 Table Correlation analysis of tissue protein levels and time from index surgery to revision surgery in TKA patients with aseptic loosening (AL) and no clinical/radiographic signs of AL (non-AL) stages. Positive/Negative correlation: the levels of proteins in tissue lysates increases/decreases with time prosthesis implantation lifetime (time from primary to revision surgery).
(DOCX)

S1 Fig Protein levels of deregulated proteins differentially expressed in tissues from TKA patients. Protein levels of top-deregulated proteins in pseudosynovial membrane lysates from patients with aseptic loosening (AL, yellow dots/columns) and non-aseptic loosening (non-AL, green dots/columns) stages (left panel) and its relationship with implant lifetime (middle/right panel) are presented. The y-axis represents the normalized protein expression. The x-axis represents the implant lifetime in months from index surgery. Horizontal bars indicate group means, and diagonal bars indicate the trend of protein level changes over time; error bars indicate 95% confidence interval.
(DOCX)

S2 Fig Correlation of the levels of sTIE2, sVEGFR2, PGF, sHGF, sE-selectin and CXCL10 proteins in tissues of TKA patients with the implant lifetime. Green dots represent individual patients with no aseptic loosening (non-AL) and yellow dots with aseptic loosening (AL). The y-axis represents the normalized protein expression. The x-axis represents the lifetime of prosthesis (from index surgery to revision surgery) in months.
(DOCX)

S1 Text Immunohistochemistry and used antibodies. (DOCX)
