# Supplementary material for: Inflammation time-axis in aseptic loosening of total knee arthroplasty: A preliminary study
Source: PLoS One. 2019 Aug 30;14(8):e0221056. doi: 10.1371/journal.pone.0221056 (PMC6716666; doi:10.1371/journal.pone.0221056)
Supplement: S1 Table — (DOCX) [file pone.0221056.s001.docx]

**S1 Table. List of investigated proteins.**

| *Protein symbol* | *Protein name* |
| --- | --- |
| ADM | Adrenomedullin |
| sAREG | Amphiregulin, soluble |
| sBAFF (TNFSF13B) | B-cell activating factor, soluble |
| sBTC | Betacellulin |
| CA125 | Cancer antigen 125 |
| CA242 | CA 242 tumor marker |
| sCAIX | Carbonic anhydrase 9, soluble |
| CASP3 | Caspase-3 |
| sCathepsin D (CTSD) | Cathepsin D, soluble |
| CCL2/MCP1 | Chemokine (C-C motif) ligand 2/monocyte chemotactic protein 1 |
| CCL19 | Chemokine (C-C motif) ligand 19 |
| CCL21 | Chemokine (C-C motif) ligand 21 |
| CCL24 | Chemokine (C-C motif) ligand 24 |
| sCD30L (TNFSF8) | CD30 ligand, soluble |
| sCD40L (TNFSF5) | CD40 ligand, soluble |
| sCD69 | Cluster of Differentiation 69 (Early activation antigen CD69), soluble |
| CEA | Carcinoembryonic antigen |
| CSF1 (M-CSF) | Colony stimulating factor 1 |
| CXCL5 | C-X-C motif chemokine 5 |
| CXCL9 | Chemokine (C-X-C motif) ligand 9 |
| CXCL10 | C-X-C motif chemokine 10 |
| CXCL11 | C-X-C motif chemokine 11 |
| CXCL13 | Chemokine (C-X-C motif) ligand 13 |
| Cystatin B | Cystatin B |
| sE selectin (CD62E) | E-selectin, soluble |
| sEGF | Epidermal growth factor , soluble |
| sEGFR | Epidermal growth factor receptor, soluble |
| sEMMPRIN | Extracellular matrix metalloproteinase inducer, soluble |
| sEpCAM | Epithelial cell adhesion molecule , soluble |
| sEpiregulin (EPR) | Epiregulin, soluble |
| EPO | Erythopoietin |
| sER | Estrogen receptor, soluble |
| FABP4 | Fatty acid binding protein 4 |
| sFas (TNFRSF6) | Fas receptor, soluble |
| sFasL | Fas ligand, soluble |
| Flt3L | FMS-like tyrosine kinase 3 ligand |
| Follistatin (FS) | Follistatin |
| Galectin 3 | Galectin-3 |
| GDF15 | Growth differentiation factor 15 |
| GM-CSF (CSF2) | Granulocyte-macrophage colony-stimulating factor |
| sFOLR1 | Folate receptor alpha, soluble |
| sHBEGF | Heparin-binding EGF-like growth factor, soluble |
| HE4 | Human Epididymis Protein 4 |
| sHER2/neu (ERBB2) | Receptor tyrosine-protein kinase erbB-2, soluble |
| sHER3 (ERBB3) | Receptor tyrosine-protein kinase erbB-3, soluble |
| sHER4 (ERBB4) | Receptor tyrosine-protein kinase erbB-4, soluble |
| sHGF | Hepatocyte growth factor/scatter factor, soluble |
| sHGFR | Hepatocyte growth factor receptor, soluble |
| hGH | Human Growth Hormone |
| IFNγ | Interferon gamma |
| IL1RA | Interleukin-1 receptor antagonist |
| IL2 | Interleukin 2 |
| sIL2RA | Interleukin-2 receptor alpha chain, soluble |
| IL4 | Interleukin 4 |
| IL6 | Interleukin 6 |
| sIL6R | Interleukin 6 receptor, soluble |
| IL7 | Interleukin 7 |
| IL8 (CXCL8) | Interleukin 8 |
| IL12 | Interleukin 12 |
| sIL17RB | Interleukin-17 receptor B, soluble |
| KLK6 | Kallikrein-6 |
| KLK11 | Kallikrein-11 |
| MIA | Melanoma-derived growth regulatory protein |
| sMICA | MHC class I polypeptide-related sequence A, soluble |
| MDK (NEGF2)  MMP3 | Midkine; neurite growth-promoting factor 2  Matrix metalloproteinase-3 |
| MPO | Myeloperoxidase |
| MYD88 | Myeloid differentiation primary response 88 |
| OPG (TNFRSF11B) | Osteoprotegerin |
| sPDGFB | Platelet-derived growth factor subunit B, soluble |
| sPECAM1 | Platelet endothelial cell adhesion molecule, soluble |
| PGF | Placental growth factor |
| PRL | Prolactin |
| PRSS8 | Prostasin |
| PSA | Prostate-specific antigen |
| REG4 | Regenerating islet-derived protein 4 |
| sSCF | Stem cell factor, soluble |
| sTF | Tissue Factor, soluble |
| sTGFA | Transforming growth factor alpha, soluble |
| TGFB1 | Transforming growth factor beta 1 |
| THPO | Thrombopoietin |
| sTIE2 | Receptor tyrosine kinase Tie2, soluble |
| TNFα | Tumor necrosis factor alpha |
| sTNFR1 (TNFRSF1A) | Tumor necrosis factor receptor 1, soluble |
| sTNFR2 (TNFRSF1B) | Tumor necrosis factor receptor 2, soluble |
| sTNFRSF4 (OX40) | Tumor necrosis factor receptor superfamily member 4, soluble |
| TNFSF14 (LIGHT) | Tumor necrosis factor superfamily member 14 |
| TRAP | Tartrate-resistant acid phosphatase |
| suPAR | Urokinase plasminogen activator receptor, soluble |
| sVEGFA | Vascular endothelial growth factor A, soluble |
| sVEGFD | Vascular endothelial growth factor D, soluble |
| sVEGFR2 | Vascular endothelial growth factor receptor 2, soluble |
