# Supplementary material for: Inflammation time-axis in aseptic loosening of total knee arthroplasty: A preliminary study
Source: PLoS One. 2019 Aug 30;14(8):e0221056. doi: 10.1371/journal.pone.0221056 (PMC6716666; doi:10.1371/journal.pone.0221056)
Supplement: S2 Table — (DOCX) [file pone.0221056.s002.docx]

**S2 Table. Protein levels in tissue lysates from TKA patients with/without aseptic loosening (AL / non-AL) of TKA.**

|  | | | | | | | | |  |
| --- | --- | --- | --- | --- | --- | --- | --- | --- | --- |
| *Analyte* | *Mean Linear ddCq (95% CI)* | | *FC* | | *P* | | *P_corr_* | |  |
|  | ***Non-AL*** | ***AL*** | |  |  | |  | |  |
| sTNFR2 | 8.2 (4.7-11.7) | 35.7 (21.9-49.5) | | 4.47 | 8.165 × 10^-5^ | | 6.70 × 10^-3^ | |  |
| sAREG | 1.5 (1.0-2.1) | 4.4 (3.1-5.7) | | 3.65 | 6.600 × 10^-4^ | | | 2.46 × 10^-2^ |  |
| IL8 | 4,225 (997-7,453) | 23,758 (14,991-32,524) | | 5.68 | 9.390 × 10^-4^ | | | 2.46 × 10^-2^ |  |
| CSF1 | 94.8 (55.2-134.4) | 259.7 (161.5-357.9) | | 2.71 | 1.306 × 10^-3^ | | | 2.46 × 10^-2^ |  |
| sFasL | 26.5 (16.7-36.2) | 51.4 (39.4-63.3) | | 2.21 | 1.803 × 10^-3^ | | | 2.46 × 10^-2^ |  |
| sIL6R | 0.022 (0.010-0.034) | 0.067 (0.043-0.090) | | 3.89 | 1.803 × 10^-3^ | | | 2.46 × 10^-2^ |  |
| MPO | 4.7 (2.7-6.7) | 16.0 (8.9-23.1) | | 3.12 | 2.436 × 10^-3^ | | | 2.50 × 10^-2^ |  |
| TNFSF14 | 21.0 (11.5-30.6) | 69.1 (37.1-101.0) | | 2.84 | 2.436 × 10^-3^ | | | 2.50 × 10^-2^ |  |
| sBAFF | 8.6 (3.1-14.0) | 23.9 (14.3-33.5) | | 3.85 | 3.260 × 10^-3^ | | | 2.97 × 10^-2^ |  |
| CCL2/MCP1 | 11,257 (6,979-15,535) | 24,218 (16,709-31,728) | | 1.68 | 4.300 × 10^-3^ | | | 3.53 × 10^-2^ |  |
| KLK6 | 4.2 (2.2-6.2) | 9.8 (6.6-12.9) | | 3.33 | 5.620 × 10^-3^ | | | 4.19 × 10^-2^ |  |
| IL1RA | 2,489 (1,736-3,242) | 4,519 (3,306-5,732) | | 1.68 | 7.253 × 10^-3^ | | | 4.58 × 10^-2^ |  |
| suPAR | 40.0 (26.4-53.5) | 68.4 (51.5-85.3) | | 2.09 | 7.253 × 10^-3^ | | | 4.58 × 10^-2^ |  |
| IFNγ | 0.038 (0.027-0.050) | 0.070 (0.047-0.093) | | 1.95 | 0.012 | | | 6.43 × 10^-2^ |  |
| TRAP | 1,1367 (577-1,696) | 2,431 (1,606-3,256) | | 2.14 | 0.012 | | | 6.43 × 10^-2^ |  |
| sCD30L | 33.0 (20.2-45.8) | 57.8 (42.1-73.6) | | 2.26 | 0.015 | | | 6.73 × 10^-2^ |  |
| sIL17RB | 0.031 (0.017-0.045) | 0.062 (0.041-0.083) | | 2.68 | 0.015 | | | 6.73 × 10^-2^ |  |
| MYD88 | 8.5 (4.1-12.9) | 13.4 (9.9-16.9) | | 2.17 | 0.015 | | | 6.73 × 10^-2^ |  |
| sCD69 | 113.9 (40.3-187.6) | 240.0 (140.4-339.6) | | 2.78 | 0.023 | | | 8.87 × 10^-2^ |  |
| IL6 | 272.4 (61.6-483.1) | 1,242 (375-2,109) | | 3.89 | 0.023 | | | 8.87 × 10^-2^ |  |
| sTNFR1 | 82.7 (36.2-129.2) | 139.7 (104.4-175.1) | | 2.21 | 0.023 | | | 8.87 × 10^-2^ |  |
| CXCL10 | 2,151 (-6967-4,999) | 2,909 (1,785-4,033) | | 2.67 | 0.028 | | | 9.51 × 10^-2^ |  |
| sMICA | 52.3 (19.6-85.1) | 103.2 (63.4-143.0) | | 2.18 | 0.028 | | | 9.51 × 10^-2^ |  |
| PRL | 1.4 (0.7-2.2) | 3.0 (1.9-4.2) | | 3.07 | 0.028 | | | 9.51 × 10^-2^ |  |
| sBTC | 249.3 (136.8-361.8) | 394.7 (281.2-508.1) | | 2.01 | 0.034 | | | 0.100 |  |
| sTGFA | 5.3 (1.5-9.1) | 8.1 (5.8-10.5) | | 2.20 | 0.034 | | | 0.100 |  |
| sTNFRSF4 | 2.7 (1.4-3.9) | 4.3 (3.0-5.6) | | 1.87 | 0.034 | | | 0.100 |  |
| CXCL9 | 59.4 (-7.7-126.4) | 125.3 (42.1-208.4) | | 3.35 | 0.041 | | | 0.100 |  |
| sE selectin | 0.8 (0.3-1.2) | 1.7 (0.9-2.4) | | 3.11 | 0.041 | | | 0.100 |  |
| Galectin 3 | 16.2 (8.1-24.4) | 29.9 (19.4-40.5) | | 2.65 | 0.041 | | | 0.100 |  |
| PRSS8 | 1.3 (0.6-2.0) | 2.5 (1.4-3.6) | | 2.04 | 0.041 | | | 0.100 |  |
| THPO | 0.087 (0.047-0.100) | 0.200 (0.100-0.300) | | 2.50 | 0.041 | | | 0.100 |  |
| TGFB1 | 15.7 (10.0-21.5) | 28.4 (17.9-38.8) | | 1.52 | 0.049 | | | 0.120 |  |
| GM-CSF | 19.6 (13.0-26.2) | 28.9 (21.1-36.8) | | 1.54 | 0.058 | | | 0.140 |  |
| REG4 | 8.9 (4.6-13.1) | 15.5 (10.2-20.8) | | 2.19 | 0.058 | | | 0.140 |  |
| CXCL11 | 2.4 (0.8-3.9) | 5.4 (2.5-8.2) | | 2.76 | 0.069 | | | 0.150 |  |
| sEpiregulin | 40.5 (11.0-70.1) | 68.8 (48.6-89.1) | | 3.15 | 0.069 | | | 0.150 |  |
| sTIE2 | 7.3 (3.1-11.5) | 10.2 (7.2-13.1) | | 1.68 | 0.069 | | | 0.150 |  |
| sVEGFD | 1.2 (0.5-1.8) | 2.2 (1.4-2.9) | | 2.95 | 0.069 | | | 0.150 |  |
| sHER4 | 0.3 (0.1-0.6) | 0.6 (0.4-0.9) | | 3.00 | 0.082 | | | 0.160 |  |
| sPECAM1 | 0.6 (0.4-0.8) | 0.9 (0.6-1.1) | | 1.48 | 0.082 | | | 0.160 |  |
| sSCF | 56.5 (29.6-83.4) | 86.4 (60.1-112.7) | | 1.65 | 0.082 | | | 0.160 |  |
| HE4 | 61.8 (27.2-96.4) | 120.1 (72.9-167.3) | | 2.54 | 0.095 | | | 0.160 |  |
| IL2 | 3.7 (1.5-5.9) | 6.1 (4.2-8.1) | | 2.10 | 0.095 | | | 0.160 |  |
| IL4 | 0.3 (0.1-0.5) | 0.6 (0.4-0.8) | | 2.25 | 0.095 | | | 0.160 |  |
| IL7 | 3.5 (1.1-6.0) | 6.5 (4.4-8.6) | | 3.47 | 0.095 | | | 0.160 |  |
| sTF | 407.2 (161.8-652.6) | 558.2 (402.6-713.8) | | 1.94 | 0.095 | | | 0.160 |  |
| sVEGFR2 | 78.4 (40.5-116.3) | 113.9 (86.0-141.8) | | 1.83 | 0.095 | | | 0.160 |  |
| CCL19 | 72.2 (-26.75-171.1) | 136.8 (26.7-247.0) | | 1.82 | 0.111 | | | 0.170 |  |
| CXCL5 | 69.4 (5.0-133.8) | 183.9 (49.8-317.9) | | 4.33 | 0.111 | | | 0.170 |  |
| sEpCAM | 28.7 (4.5-52.9) | 35.2 (23.2-47.2) | | 1.93 | 0.111 | | | 0.170 |  |
| sHGF | 111.0 (48.9-173.2) | 171.0 (115.1-226.8) | | 1.82 | 0.111 | | | 0.170 |  |
| MDK | 50.8 (8.4-93.1) | 111.3 (58.5-164.1) | | 4.97 | 0.111 | | | 0.170 |  |
| CCL24 | 619 (111-1,128) | 1,124 (554-1,695) | | 1.83 | 0.129 | | | 0.180 |  |
| FABP4 | 16.9 (2.9-30.9) | 31.1 (14.2-48.1) | | 2.12 | 0.129 | | | 0.180 |  |
| GDF15 | 10.5 (5.2-15.8) | 16.4 (10.5-22.3) | | 1.44 | 0.129 | | | 0.180 |  |
| TNFα | 1.4 (0.1-2.7) | 2.9 (1.7-4.0) | | 4.97 | 0.129 | | | 0.180 |  |
| sHER3 | 51.1 (21.8-80.4) | 81.6 (53.7-109.4) | | 2.56 | 0.169 | | | 0.240 |  |
| CASP3 | 155 (105-204) | 208 (145-271) | | 1.57 | 0.193 | | | 0.260 |  |
| OPG | 661 (254-1,069) | 1,238 (562-1,913) | | 2.55 | 0.193 | | | 0.260 |  |
| CCL21 | 28.2 (4.3-52.1) | 15.5 (1.2-29.8) | | 0.84 | 0.219 | | | 0.290 |  |
| sFas | 217 (2.9-431) | 225 (150-300) | | 2.04 | 0.247 | | | 0.330 |  |
| sVEGFA | 936 (-328.1-2,199) | 811.5 (44.4-1,579) | | 2.69 | 0.277 | | | 0.360 |  |
| sEGFR | 53.0 (0.2-105.7) | 54.3 (33.9-74.6) | | 2.12 | 0.310 | | | 0.390 |  |
| sEMMPRIN | 229 (156-302) | 300 (208-392) | | 1.29 | 0.310 | | | 0.390 |  |
| ADM | 16.7 (5.5-27.9) | 24.4 (10.4-38.4) | | 1.73 | 0.345 | | | 0.400 |  |
| sEGF | 4.8 (2.2-7.3) | 5.7 (3.8-7.6) | | 1.28 | 0.345 | | | 0.400 |  |
| Follistatin | 0.5 (0.3-0.8) | 0.4 (0.2-0.6) | | 0.64 | 0.345 | 0.400 | | | |
| IL12 | 2.2 (0.8-3.6) | 2.9 (1.5-4.4) | | 1.39 | 0.345 | 0.400 | | | |
| sIL2RA | 749.7 (193.2-1,306.1) | 814.5 (539.1-1,089.8) | | 1.31 | 0.345 | 0.400 | | | |
| sPDGFB | 29.6 (11.5-47.8) | 34.4 (22.1-46.7) | | 1.23 | 0.345 | 0.400 | | | |
| sHGFR | 203.0 (85.1-321.0) | 240.4 (149.1-331.7) | | 1.76 | 0.464 | 0.530 | | | |
| Flt3L | 13.1 (4.5-21.8) | 17.5 (10.1-24.9) | | 1.60 | 0.508 | 0.570 | | | |
| sCD40L | 10.7 (1.8-19.7) | 7.1 (4.7-9.6) | | 0.83 | 0.602 | 0.660 | | | |
| PGF | 1,150.3 (373.3-1,927.2) | 1,222.2 (705.1-1,739.3) | | 1.20 | 0.602 | 0.660 | | | |
| sHBEGF | 12.5 (7.5-17.5) | 11.7 (7.0-16.4) | | 1.01 | 0.754 | 0.810 | | | |
| sCathepsin D | 6.5 (2.9-10.0) | 7.5 (4.1-10.8) | | 1.22 | 0.808 | 0.850 | | | |
| hGH | 16.0 (5.0-27.0) | 18.9 (5.5-32.3) | | 0.71 | 0.808 | 0.850 | | | |
| CXCL13 | 121.0 (7.8-234.1) | 96.3 (41.5-151.1) | | 0.56 | 0.917 | 0.950 | | | |
| sHER2/neu | 1.8 (0.4-3.3) | 1.4 (0.9-1.9) | | 0.94 | 0.972 | 0.980 | | | |
| sFOLR1 | 1.0 (0.5-1.5) | 1.0 (0.6-1.4) | | 0.96 | 0.972 | 0.980 | | | |
| Cystatin B | 279.8 (141.1-418.4) | 312.1 (156.5-467.6) | | 1.21 | 1.000 | 1.000 | | | |

FC: fold change

All the data are presented as Mean Expression Level (95% CI).

*P* value corr: value corrected for multiple comparisons (Benjamini-Hochberg correction)
