# Supplementary material for: Inflammation time-axis in aseptic loosening of total knee arthroplasty: A preliminary study
Source: PLoS One. 2019 Aug 30;14(8):e0221056. doi: 10.1371/journal.pone.0221056 (PMC6716666; doi:10.1371/journal.pone.0221056)
Supplement: S3 Table — Positive/Negative correlation: the levels of proteins in tissue lysates increases/decreases with time prosthesis implantation lifetime (time from primary to revision surgery). (DOCX) [file pone.0221056.s003.docx]

**S3 Table.** **Correlation analysis of tissue protein levels and time from index surgery to revision surgery in TKA patients with aseptic loosening (AL) and no clinical/radiographic signs of AL (non-AL) stages.**

Positive/Negative correlation: the levels of proteins in tissue lysates increases/decreases with time prosthesis implantation lifetime (time from primary to revision surgery).

| ***Protein*** | ***Spearman rank-order coefficient*** *(r_s_)* | ***P value*** |
| --- | --- | --- |
| ***Whole cohort of TKA patients*** | | |
| sTNFR2 | 0.76 | 6.15 x 10^-5^ |
| IL8 | 0.73 | 1.76 x 10^-4^ |
| TRAP | 0.65 | 1.56 x 10^-3^ |
| sCD69 | 0.61 | 3.17 x 10^-3^ |
| MPO | 0.60 | 3.70 x 10^-3^ |
| sAREG | 0.60 | 4.09 x 10^-3^ |
| IL1RA | 0.59 | 4.52 x 10^-3^ |
| sIL6R | 0.59 | 4.68 x 10^-3^ |
| CCL2/MCP1 | 0.58 | 5.47 x 10^-3^ |
| TNFSF14 | 0.58 | 6.15 x 10^-3^ |
| IFNγ | 0.57 | 6.67 x 10^-3^ |
| CSF1 | 0.53 | 1.26 x 10^-2^ |
| sFasL | 0.53 | 1.37 x 10^-2^ |
| sIL17RB | 0.53 | 1.38 x 10^-2^ |
| sBAFF | 0.50 | 2.14 x 10^-2^ |
| sPECAM1 | 0.47 | 3.24 x 10^-2^ |
| suPAR | 0.46 | 3.61 x 10^-2^ |
| Galectin 3 | 0.45 | 4.14 x 10^-2^ |
| CCL19 | 0.45 | 4.31 x 10^-2^ |
| KLK6 | 0.44 | 4.85 x 10^-2^ |
| CCL21 | -0.45 | 4.05 x 10^-2^ |
| ***Without aseptic loosening (non-AL)*** | | |
| sTIE2 | -0.85 | 3.42 x 10^-3^ |
| sVEGFR2 | -0.83 | 5.80 x 10^-3^ |
| PGF | -0.79 | 1.19 x 10^-2^ |
| sE selectin | -0.70 | 3.47 x 10^-2^ |
| sHGF | -0.69 | 4.12 x 10^-2^ |
| ***Aseptic loosening (AL)*** | | |
| CXCL10 | -0.67 | 1.71 x 10^-2^ |
