# Supplementary material for: Inflammation time-axis in aseptic loosening of total knee arthroplasty: A preliminary study
Source: PLoS One. 2019 Aug 30;14(8):e0221056. doi: 10.1371/journal.pone.0221056 (PMC6716666; doi:10.1371/journal.pone.0221056)

**S1 Fig. Protein levels of deregulated proteins differentially expressed in tissues from TKA patients.**

Protein levels of top-deregulated proteins in pseudosynovial membrane lysates from patients with aseptic loosening (AL, yellow dots/columns) and non-aseptic loosening (non-AL, green dots/columns) stages (left panel) and its relationship with implant lifetime (middle/right panel) are presented. The y-axis represents the normalized protein expression. The x-axis represents the implant lifetime in months from index surgery. Horizontal bars indicate group means, and diagonal bars indicate the trend of protein level changes over time; error bars indicate 95% confidence interval.


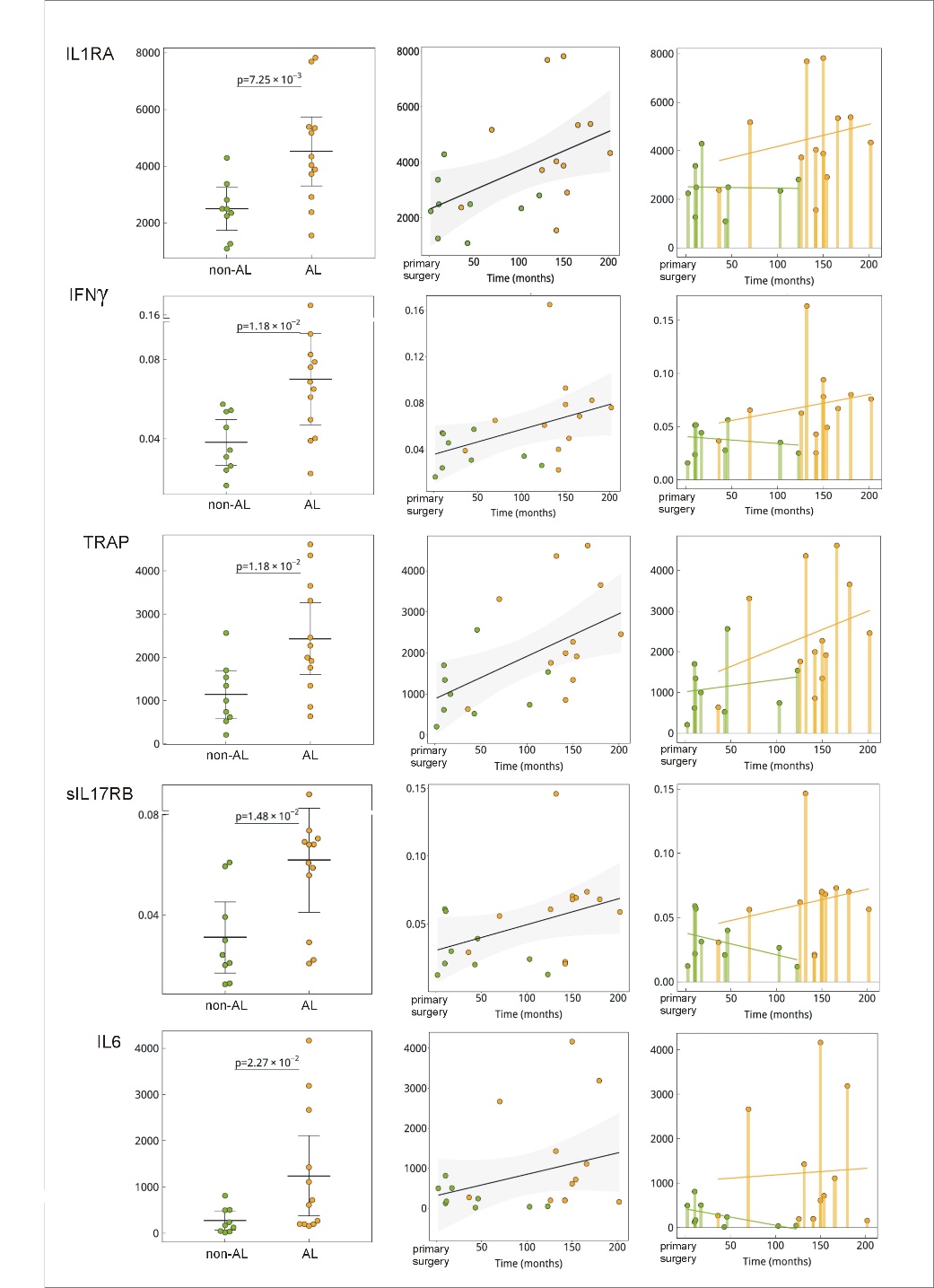


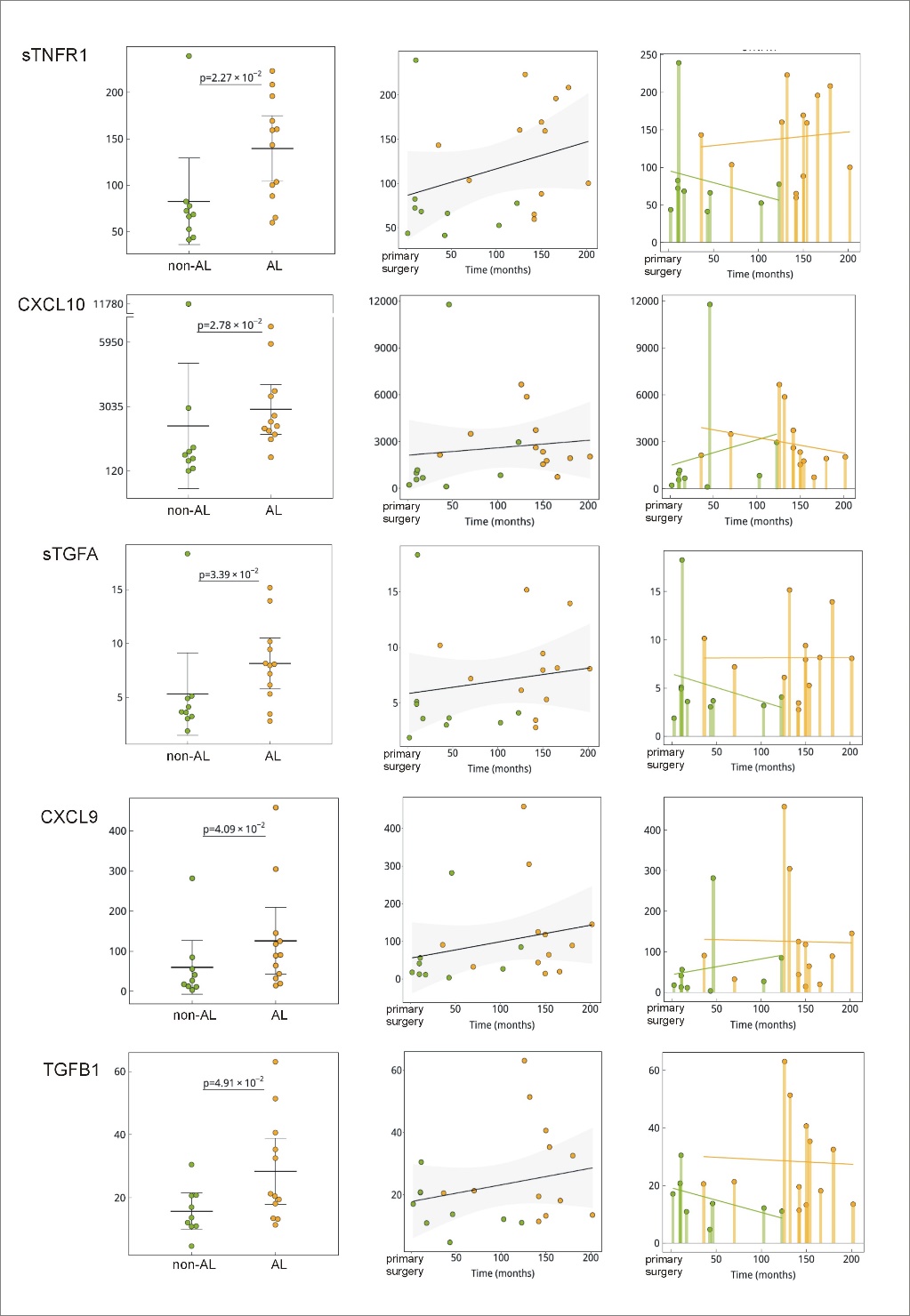

Supplement: S1 Fig — Protein levels of top-deregulated proteins in pseudosynovial membrane lysates from patients with aseptic loosening (AL, yellow dots/columns) and non-aseptic loosening (non-AL, green dots/columns) stages (left panel) and its relationship with implant lifetime (middle/right panel) are presented. The y-axis represents the normalized protein expression. The x-axis represents the implant lifetime in months from index surgery. Horizontal bars indicate group means, and diagonal bars indicate the trend of protein level changes over time; error bars indicate 95% confidence interval. (DOCX) [file pone.0221056.s004.docx]
