# Supplementary material for: Inflammation time-axis in aseptic loosening of total knee arthroplasty: A preliminary study
Source: PLoS One. 2019 Aug 30;14(8):e0221056. doi: 10.1371/journal.pone.0221056 (PMC6716666; doi:10.1371/journal.pone.0221056)

**S2 Fig.** **Correlation of the levels of sTIE2, sVEGFR2, PGF, sHGF, sE-selectin and CXCL10 proteins in tissues of TKA patients with the implant lifetime.**

Green dots represent individual patients with no aseptic loosening (non-AL) and yellow dots with aseptic loosening (AL). The y-axis represents the normalized protein expression. The x-axis represents the lifetime of prosthesis (from index surgery to revision surgery) in months.


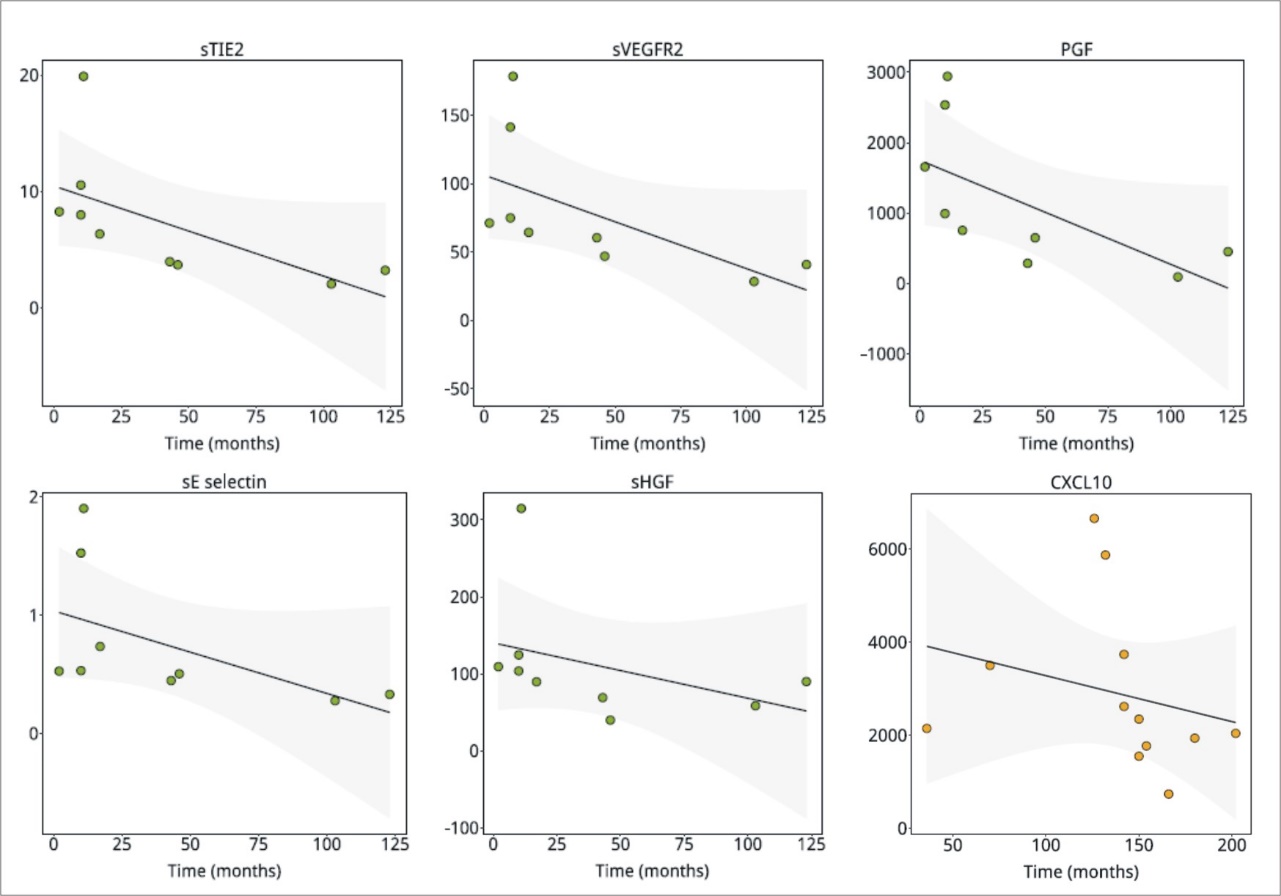

Supplement: S2 Fig — Green dots represent individual patients with no aseptic loosening (non-AL) and yellow dots with aseptic loosening (AL). The y-axis represents the normalized protein expression. The x-axis represents the lifetime of prosthesis (from index surgery to revision surgery) in months. (DOCX) [file pone.0221056.s005.docx]
