# Supplementary material for: Inflammation time-axis in aseptic loosening of total knee arthroplasty: A preliminary study
Source: PLoS One. 2019 Aug 30;14(8):e0221056. doi: 10.1371/journal.pone.0221056 (PMC6716666; doi:10.1371/journal.pone.0221056)
Supplement: S1 Text — (DOCX) [file pone.0221056.s006.docx]

**S1 Text. Immunohistochemistry and used antibodies.**

**Methods**

**Immunohistochemistry and used antibodies**

Primary antibodies were used as follows: AREG (rabbit polyclonal 1:100, Novus biologicals, Cambridge, UK), TNFR2 (rabbit polyclonal 1:100, Novus biologicals), CCL2 (rabbit polyclonal 1:500, Novus biologicals), IL8 (rabbit polyclonal 1:100, Novus biologicals), TRAP (ab49507, mouse monoclonal 1:200, Abcam, Cambridge, UK); all primary antibodies used were tested on positive control tissue specimens recommended by the manufacturer. In all cases, antigen retrieval was performed in a 10 mM sodium citrate buffer, pH 6 (120°C for 5 min) using the high-pressure histoprocessor HistosPro (Milestone, MI, USA); the EnVision^TM^ Dual Link System-HRP (Dako, Glostrup, Denmark) was used for antigen visualisation as described previously (1). The staining with a negative isotype control antibody (rabbit IgG, polyclonal – Isotype Control, 1:100, Abcam) was performed for all examined tissues.

**Semiquantitave evaluation of immunohistochemistry**

The immunohistrochemistry were calculated by semi-quantitatively using an immunohistoscore. Briefly, the percentage of strongest positive cells of each type (macrophages, giant osteoclast-like cells) in whole specimen was graded as follows: grade 0 (less than 1% of cells), grade 1 (1-30%), grade 2 (31-60%), grade 3 (more than 60%). The intensity of staining was graded as follows: grade 0 (no staining), grade 1 (weak), grade 2 (moderate), grade 3 (strong). The overall score of the expression (range 0 to 9) was obtained by multiplying the percentage by the intensity, whereas the expression with overall score i) 0 was considered as negative, ii) 1 to 3 as a weak expression, and iii) 4 to 6 as a moderate expression and iv) 7 to 9 as a strong expression. Only areas without necrosis were counted.

**References:**

**1.** Takacova S*, et al.* (2012) DNA damage response and inflammatory signaling limit the MLL-ENL-induced leukemogenesis in vivo. *Cancer cell* 21(4):517-531.
